# Hominy

**Published:** 1860-01

**Authors:** 


### HOMINY.

WHEN that kind of Indian corn called "flint corn" is broken into three or four pieces with a wooden pestle, as is done in the West and South-west, it is called hominy. The outer skin, which answers to the "bran" in grinding wheat, is removed by steeping or boiling it in the ley of wood ashes. In the North this corn broken coarsely, is called "samp," while that which is denominated "samp" in the South, is the same corn prepared in such a way that each particle as it appears on the table is not larger than a grain of rice, and is quite as white, but it has not the juiciness and sweetness of the coarser preparation.

Next to the common white bean, hominy is the most nutritious, the most economical, and the most healthful article of vegetable growth which can be placed on our tables. The usual mode of preparing it is to cover it an inch deep with water over night, and let it soak until the morning, then boil it slowly and steadily six, eight or ten hours until it is quite soft enough for being eaten easily. After it has thus been boiled, a part of it may be taken, prepared with a little milk and butter, and placed on the table, to be eaten as a vegetable or with syrup or loaf-sugar as a desert. The portion laid away can be cut in slices, about half an inch thick, and fried brown for breakfast, with or without the addition of syrup, or it may be warmed up just as it is, or with a little milk, or a tablespoonful or two in a bowl of good milk, will of itself make a sufficient meal. A bowl of milk and hominy thus prepared would make a sustaining and healthful dinner for a day laborer. If prepared fresh every day, it can be taken for weeks together with an appetite and a relish, while it is perhaps not inferior to cracked wheat as an agency in the healthful regulation of the system.

